# SCF‐FBXL8 contributes to liver metastasis and stem‐cell‐like features in colorectal cancer cells by mediating ubiquitination and degradation of TP53

**DOI:** 10.1002/ctm2.1208

**Published:** 2023-02-28

**Authors:** Jing Yao, Xin‐Ping Wang, Jun Yang, Zhe Yang, Zheng‐Yun Zhang

**Affiliations:** ^1^ Department of Surgery Shanghai Sixth People's Hospital Affiliated to Shanghai Jiao Tong University School of Medicine Shanghai China

**Keywords:** colorectal cancer, FBXL8, P53, stem‐cell, ubiquitination

## Abstract

**Background:**

FBXL8 is a conserved F‐box protein, belonging to the ubiquitin ligase complex, which promotes the development and progression of tumours. However, the regulation function and mechanism of FBXL8's involvement in colorectal cancer (CRC) remain unclear.

**Methods:**

RT–PCR is used to detect gene expression levels. Protein levels were determined by western blotting and flow cytometry. The bindings of FBXL8 and p53 and ubiquitination levels were detected by cell transfection and immunoprecipitation. The transwell assay was used to measure the ability of cells to migrate and invade. Animal studies were used to verify the function of FBXL8 in vivo.

**Results:**

The expression of FBXL8 was up‐regulated in CRC tissues, and its overexpression was associated with poor prognosis in CRC patients. The up‐regulation of FBXL8 promoted the proliferation, invasion and migration of CRC tumour cells and maintained the stem‐cell characteristics of colorectal tumour cells. Further analysis demonstrated that FBXL8 targeted p53 and reduced its stability through ubiquitination. Knockout of FBXL8 down‐regulated the proliferation, migration and stem‐like properties of tumour cells. CRC mouse xenograft tumour model confirmed that FBXL8 gene knockout inhibited tumour formation and liver metastasis.

**Conclusion:**

FBXL8 was highly expressed in CRC. Mechanism studies have shown that FBXL8 degraded tumour suppressor gene p53 by ubiquitination. FBXL8 knockout inhibited the proliferation and stem characteristics of CRC cells, so SCF‐FBXL8‐TP53 has potential to be used as a therapeutic target for CRC in subsequent studies.

## Graphical abstract

Graphical Abstract

•FBXL8 was up‐regulated in CRC tissues and associated with poor prognosis in CRC patients.

•The up‐regulation of FBXL8 promoted the proliferation, invasion, migration and maintained the stem‐cell characteristics of colorectal tumour cells.

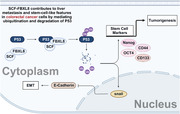

## 1 INTRODUCTION

Colorectal cancer (CRC) is one of the three cancer‐related deaths in the world.^1^, ^2^, ^3^ In recent years, due to the continuous improvement of surgical methods and the updating of radiotherapy and chemotherapy regimen, the efficacy of colon cancer treatment has been greatly improved.^4^, ^5^ The mortality and prognosis of colon cancer patients have not improved obviously.^6^, ^7^ Cancer stem cells (CSCs) are considered to be the root cause of tumour growth, metastasis and recurrence.^8^, ^9^ Studies have found that the activation and maintenance of CSC‐related pathways are closely related to the recurrence, invasion and metastasis of CRC.^10^

Tumour suppressor gene TP53 (p53) is a transcription factor which could prevent the growth as well as division of damaged and potential precancerous cells, thereby inhibiting tumourigenesis.^11^, ^12^ The expression, activity, as well as subcellular localization of p53 are all precisely regulated by multiple posttranslational modifications and therefore play an important role in tumour regulation.^13^, ^14^

Cullin‐ring ligase (CRL) is a multi‐subunit RING finger family containing E3 ligase, constituting the largest E3. Skp1‐cul1‐F‐box (SCF) E3 ligase, also known as CRL1, is the founding member and most representative of CRL.^15^ SCF E3 ligase consists of four subunits: scaffold protein CUL1, adaptor protein SKP1, an F‐box protein and F‐box proteins.^16^, ^17^ Previous studies of our group have found that FBXW11, an F‐box protein, degrades HIC1 tumour suppressor protein through SCF ubiquitin system, thereby promoting the proliferation and stem‐cell‐like characteristic of CRC cells.^18^

FBXL8 is a novel F‐box protein belonging to the FBXL family with leucine‐rich repeat sequence (LRR).^19^ FBXL8 was found to interact with two tumour suppressors, cyclin D2 (CCND2) and interferon regulatory factor 5 (IRF5). FBXL8 knockdown leads to the accumulation of CCND2 and IRF5 in breast cancer cells and inhibited tumour progression.^19^ In vitro analysis of 1349 matched breast cancer tissues showed that FBXL8 promoted cell survival and tumourigenesis, and its level increased with BRCA progression.^20^ It has also been proved that the F‐box region of FBXL8 can interact with the SCF complex, thus playing an important role in ubiquitination and degradation of downstream proteins.^21^, ^22^ However, FBXL8 has been less reported in CRC. We hypothesized that FBXL8 degrades the tumour suppressor gene P53 through ubiquitination, leading to the pathological progression of CRC. By analysing the database and our clinical specimens, knockout of FBXL8, as well as immunoprecipitation and in vivo experiments, we found that FBXL8 was significantly up‐regulated in CRC and promoted CRC cell proliferation, migration and stem‐like properties through ubiquitination degradation of the tumour suppressor gene p53.

## 2 METHODS

### 2.1 Bioinformatics analysis

We analysed the TIMER database (https://cistrome.shinyapps.io/timer/) to gain the FBXL8 expression in all cancer (CRC). UALCAN database was used to analyse the expression of FBXL8 in tumour tissues of CRC patients. Kaplan–Meier survival analysis was performed on CRC datasets in TCGA using GEPIA (http://gepia.cancer‐pku.cn/). UbiBrowser database (http://ubibrowser.ncpsb.org.cn/) can predict FBXL8 to p53 protein ubiquitin.

### 2.2 CRC patients and clinical samples

This study has been authorized by Ethics Committee of Shanghai Sixth People's Hospital Affiliated to Shanghai Jiao Tong University School of Medicine. We enrolled 100 patients with CRC, including 36 CRC patients without liver metastasis as well as 64 CRC patients with liver metastasis (mCRC). CRC tissue was collected during surgery, with one specimen fixed with 4% paraformaldehyde (Goodbio Technology, Wuhan, China) for immunohistochemical studies and the other specimen stored in liquid nitrogen for gene and protein levels.

### 2.3 Cell culture and transfection

The cells used in this study, including human normal intestinal epithelial cell lines: NCM460 and CRC cell lines (HT29, HCT116, SW48 and HCT15) and HEK293T cells, were purchased from the Cell Resource Center of Shanghai Institutes of Biological Sciences, Chinese Academy of Sciences.

Different combinations of plasmids were transfected into CRC cells or HEK293T cells using Lipofectamine 3000 (Invitrogen, Carlsbad, CA, USA). After indicated transfection time, the cells were collected for subsequent experiments.

To build FBXL8 knockout cell lines, sgRNAs against FBXL8 were cloned into lentiCRISPR v2 plasmid obtained from Santa Cruz biotechnology (SCBT, USA). The sgRNA sequences are as follows: 5′‐CACCGATGAGCGCCAACACTTCCTC‐3′ and 5′‐AAACGAGGAAGTGTTGGCGCTCATC‐3′. CRC cells were transfected with lentiCRISPR v2 containing sgRNA against FBXL8. Knockout clones were determined by western blotting after single clone isolation.

### 2.4 Haematoxylin–eosin staining (H&E) and immunohistochemistry

The fresh specimens were fixed in 4% paraformaldehyde (Goodbio Technology) for 24 h then embedded in paraffin and cut into 4 μm thick‐slices, which were dewaxed and dehydrated and stained with hematoxylin and eosin. For immunohistochemistry, tissue sections were dewaxed and dehydrated, and then the antigens were repaired by thermal repair antigen method and then incubated with specific primary antibodies. The primary antibodies included FBXL8 (Santa Cruz Biotechnology, California, USA, cat sc‐390582), p53 (Abcam, Cambridge, UK, cat ab26), CD133 (Abcam, cat ab222782) and Ki67 (Abcam, cat ab15580). For staining, scores of FBXL8 and p53 in tissues were determined as previous study.^23^

### 2.5 Real‐time quantitative PCR and western blotting

TRIzol was used to extract total RNA of cells or tissue. After the integrity was verified by electrophoresis, RNA was reversely transcribed into cDNA by reverse transcription kit (Takara, Dalian, China). SYBR Green reagent was used for qRT–PCR experiment.^24^ The primer sequence is as follows: FBXL8 5′‐ATCAGTTGCGAATGTGAGCTG‐3′ and 5′‐TCGAAGAGCGGTTTTTCTCCG‐3′.

Total cell protein from cells or tissue was extracted by total protein extraction kit (Goodbio Technology), and then protein loading buffer was added, denaturated at 95°C for 15 min and stored at −20°C. Protein was separated by 10% SDS electrophoresis and transferred onto PVDF membrane. Nonspecific antigen sites were blocked with 5% BSA and incubated for 1 h, and then membranes were incubated with primary antibodies (1:1000 dilution) for overnight at 4°C.^25^ Antibodies used were listed as follows: FBXL8 (cat sc‐390582), p53 metalloprotease 2 (MMP2, cat ab92536), MMP9 (cat ab76003), *E*‐cadherin (cat ab1416), snail (cat ab216347), Nanog (cat ab109250), octamer‐binding transcription factor 4 (OCT4, cat ab181557), CD44 (cat ab243894), SRY‐box transcription factor 2 (SOX2, cat ab92494) and CD133 (cat ab222782). All antibodies except FBXL8 were purchased from Abcam.

### 2.6 Co‐immunoprecipitation and ubiquitin analysis

The cells were collected 24 h after transfection and washed with PBS for three times. Proteins were extracted by adding 500 μL NP40 lysate containing protease inhibitor in to the cell pellet and then incubated for 20 min on ice. The mixtures were ultrasonic broken 10 times, each time interval of 2 s. Then the mixtures were centrifuged at 4°C for 10 min at 12000 r/min. Supernatant of 50 μL was used as the whole cell lysate (Input), 30 μL anti‐Flag affinity agarose beads were added to the remaining supernatant, and the cells were rotated and incubated at 4°C overnight. Centrifugation was performed at 2000 r/min at 4°C for 3 min, and the supernatant was discarded. Add 500 μL NP40 solution (Goodbio Technology), rotate for 3 min at 4°C, centrifuge at 2000 r/min at 4°C for 3 min, discard the supernatant and repeat for three times. Add 100 μL 1× SDS cracking buffer, cook the samples in metal bath for 10 min and then centrifuge the samples at 4°C and 12000 r/min for 10 min. The supernatant was analysed by SDS–PAGE and western blotting.^26^

### 2.7 Cell viability, invasion and migration

The cells in each group were digested with .25% trypsinase (Goodbio Technology) and cultured in 96‐well plates at the density of 1000 cells/well for 0, 24, 48 and 72 h, then cultured with 10 μL CCK‐8 solution (Goodbio Technology, G4103) for 4 h. The optical density of each well was detected at 450 nm with a microplate reader.

For detecting cell proliferation, 100 μL EDU solution was added to each well, and the cells were incubated for 2 h. The cells were fixed with paraformaldehyde and then stained with Apollo 567 as well as Hoechst 33342 under dark conditions. The number of EDU positive cells was detected by an inverted fluorescence microscope and photographed, and the ratio of Apollo 567 positive cells to Hoechst 33342 labelled cells was taken as EdU positive cell rate.

For the cell migration experiment, 1 × 10^5^ cell suspension (200 μL) was inoculated into the upper chamber of transwell, followed by 800 μL DMEM medium (Gibco, Grand Island, NY, USA) containing 10% foetal bovine serum in the lower chamber. After 24 h of culture, upper were wiped with cotton swabs, followed by formaldehyde fixation and crystal violet staining. In order to test the invasion ability of cells, matrix glue was prelaid in the upper chamber of transwell before cell suspension was added, and the subsequent experimental steps were the same as the migration experiment.^27^ Five fields were randomly selected under an optical microscope to observe, count the number of migrating or invading cells and take photos.

### 2.8 Sphere formation

The cells were cultured for 10 days as previously stated.^28^ In brief, 500 cells/wells were seeded into 6‐well ultra‐low cluster plates (Corning, NY, USA). Cells were cultured in DMEM, including 2% B27 (Invitrogen), 20 ng/mL human EGF (Sigma‐Aldrich, St. Louis, MO, USA), 20 ng/mL human bFGF (Sigma‐Aldrich), 5 μg/mL insulin (Sigma‐Aldrich) and .4% BSA (Sigma‐Aldrich). After 10 days of culture, an optical microscope was used to counted microspheres with larger than 100 μm in diameter.

### 2.9 Flow cytometry

The transfected HT29 and HCT116 cells were digested into single cells with .25% trypsin, then the anti‐ALDH1‐APC antibody (BioLegend, San Diego, CA, USA) was incubated with cells for 20 min under dark conditions, and the number of ALDH1‐positive cells was analysed by flow cytometry.

### 2.10 In vivo experiment

Male BABL/C female nude mice (4–6‐week old) were obtained from the Animal Center of Guangdong province (Guangzhou, China) to use for in vivo assay. Animal experiments have been approved by Ethics Commitee of Shanghai Sixth People's Hospital Affiliated to Shanghai Jiao Tong University School of Medicine. In order to explore the effect of FBXL8 on CRC, 12 BALB/C nude mice were divided into two groups with 6 mice in each group, and the xenograft tumour model was established by subcutaneous injection of HT29 (5 × 10^5^ cells). After 8 weeks, the mice were sacrificed.

In order to explore the effect of FBXL8 on liver metastasis of CRC, 12 BALB/C nude mice were divided into two groups with 6 cells in each group. HT29 cells (5 × 10^5^) were injected into the spleen of mice to establish tumour metastasis model.^29^ Liver metastasis was detected by IVIS Lumina imaging system.^30^ In brief, HT29‐Luc cells transfected with FBXL8 KO or FBXL8 WT were injected into the spleen of mice. At the end of 6 weeks, after intraperitoneal injection of d‐luciferin, all mice were imaged using the Xenogen IVIS Spectrum Imaging System (Caliper Life Sciences, USA) before being sacrificed. The liver tissues of mice were taken for paraffin embedding and then HE staining to detect the number of liver metastatic lymph nodes.

### 2.11 Statistical analysis

SPSS 22.0 statistical software was used for statistical test. The data were expressed as mean ± standard deviation. Kaplan–Meier was used to analyse survival rate. Correlation analysis was performed by spearman test. Two samples *t* test was used for comparison between groups, and *p* < .05 was considered statistically significant. Image data were collected and processed by Graphpad Prism 7.0 (GraphPad Software, La Jolla, CA, USA). Each cell experiment was repeated three times, and animal experiments were repeated six times.

## 3 RESULTS

### 3.1 FBXL8 was up‐regulated in CRC tissue and CRC cell lines

**FIGURE 1:**
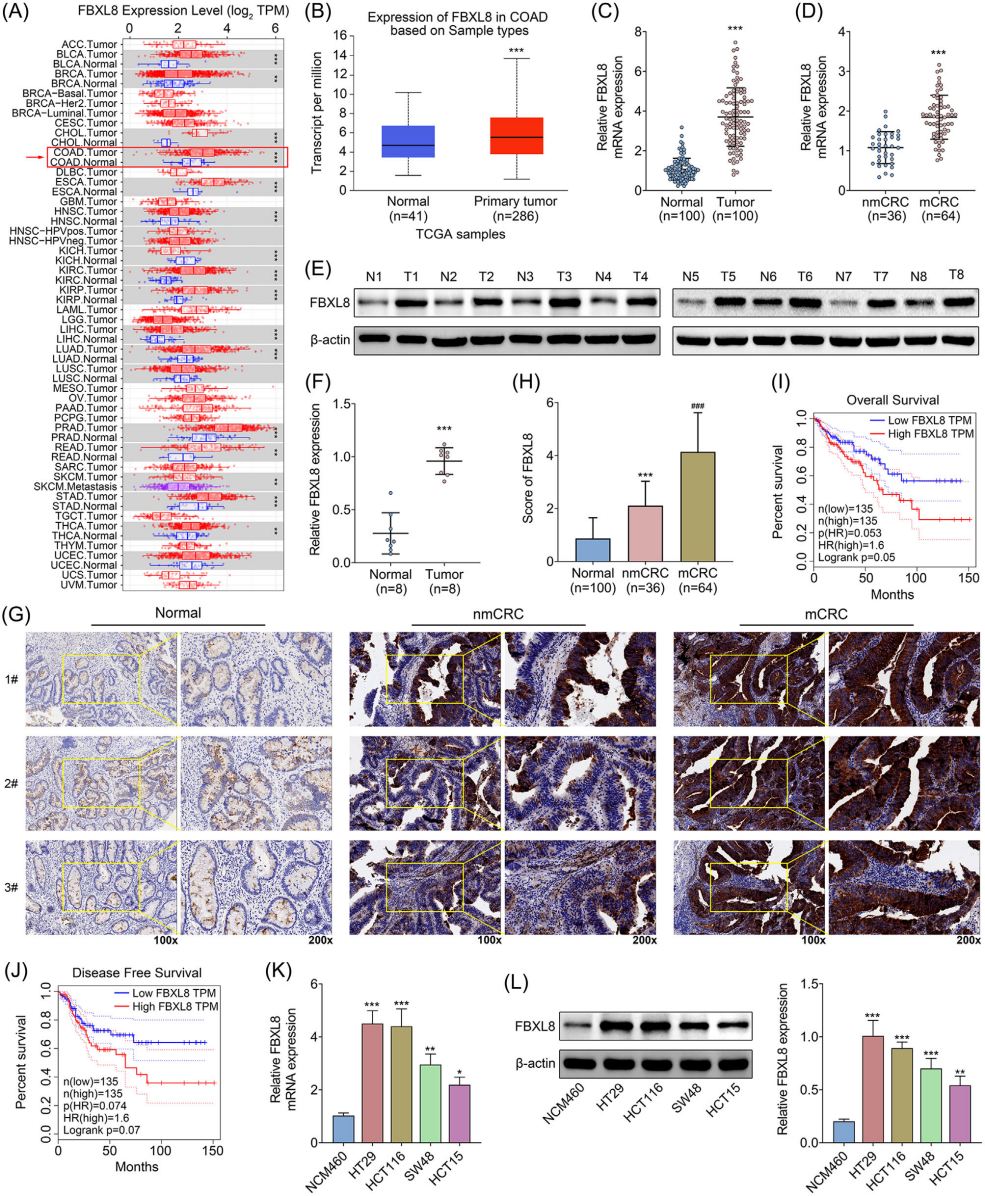
FBXL8 is up‐regulated in colorectal cancer (CRC) tissue and CRC cell lines. (A) Expression of FBXL8 in various tumour tissues. (B) The expression levels of FBXL8 in CRC patients in TCGA database. (C) The relative mRNA expression level FBXL8 in CRC tissues and normal paracancer tissues. (D) The relative mRNA expression level FBXL8 in nmCRC and mCRC groups. (E and F) The protein levels of FBXL8 in CRC tissues and normal adjacent tissues were detected by western blotting. (G) Representative immunohistochemistry images showing FBXL8 in the normal group, nmCRC and mCRC tissues. (H) Normal group, nmCRC and mCRC tissues were scored based on FBXL8 immunohistochemical expression levels. Overall survival (I) and disease‐free survival (J) were analysed by Kaplan–Meier curve. FBXL8 was analysed in human normal intestinal epithelial cell lines: NCM460 and CRC cell lines (HT29, HCT116, SW48 and HCT15). The relative mRNA (K) and protein (L) expression levels of NCM460 and CRC cell lines were detected by RT–PCR and western blotting, respectively. *p < .05, **p < .01, ***p < .001 compared with normal group.

Based on the TIMER database, we analysed that FBXL8 was expressed in a variety of tumours, including CRC (Figure 1A), and the expression of FBXL8 in CRC was significantly increased (Figure 1B). By analysing our clinical samples, RT–PCR results showed that CRC tissues showed significantly higher expression levels of FBXL8 compared to normal paracancer tissues (Figure 1C). Further, CRC was divided into the non‐liver metastasis group (nmCRC) and the presence of liver metastasis group (mCRC), and the expression levels of FBXL8 were higher in the mCRC group than in the nmCRC group (Figure 1D). The higher protein expression level of FBXL8 in CRC tissues was confirmed by western blotting assay (Figure 1E,F). Then, through immunohistochemical analysis of the expression levels of FBXL8 in the normal group, nmCRC and mCRC groups, we found that the FBXL8 positive cells in nmCRC and mCRC groups were significantly increased (Figure 1G). Moreover, the score of FBXL8 in nmCRC and mCRC groups was significantly higher than that in the normal group (Figure 1H). Overall survival and disease‐free survival of CRC patients with high FBXL8 were found to be lower than CRC patients with low FBXL8 (Figure 1I,J). In addition, we detected the expression levels of FBXL8 in human normal intestinal epithelial cell lines: NCM460 and CRC cell lines (HT29, HCT116, SW48 and HCT15). The mRNA and protein levels of FBXL8 were higher in CRC cell lines compared with normal epithelial cell line by RT–PCR and western blotting, respectively (Figure 1K,L). As FBXL8 was significantly overexpressed in HT29 and HCT116 cells, subsequent studies were conducted with HT29 and HCT116 cells. These data suggest that FBXL8 is up‐regulated in CRC tissues and cell lines.

### 3.2 FBXL8 promoted ubiquitination of p53

**FIGURE 2:**
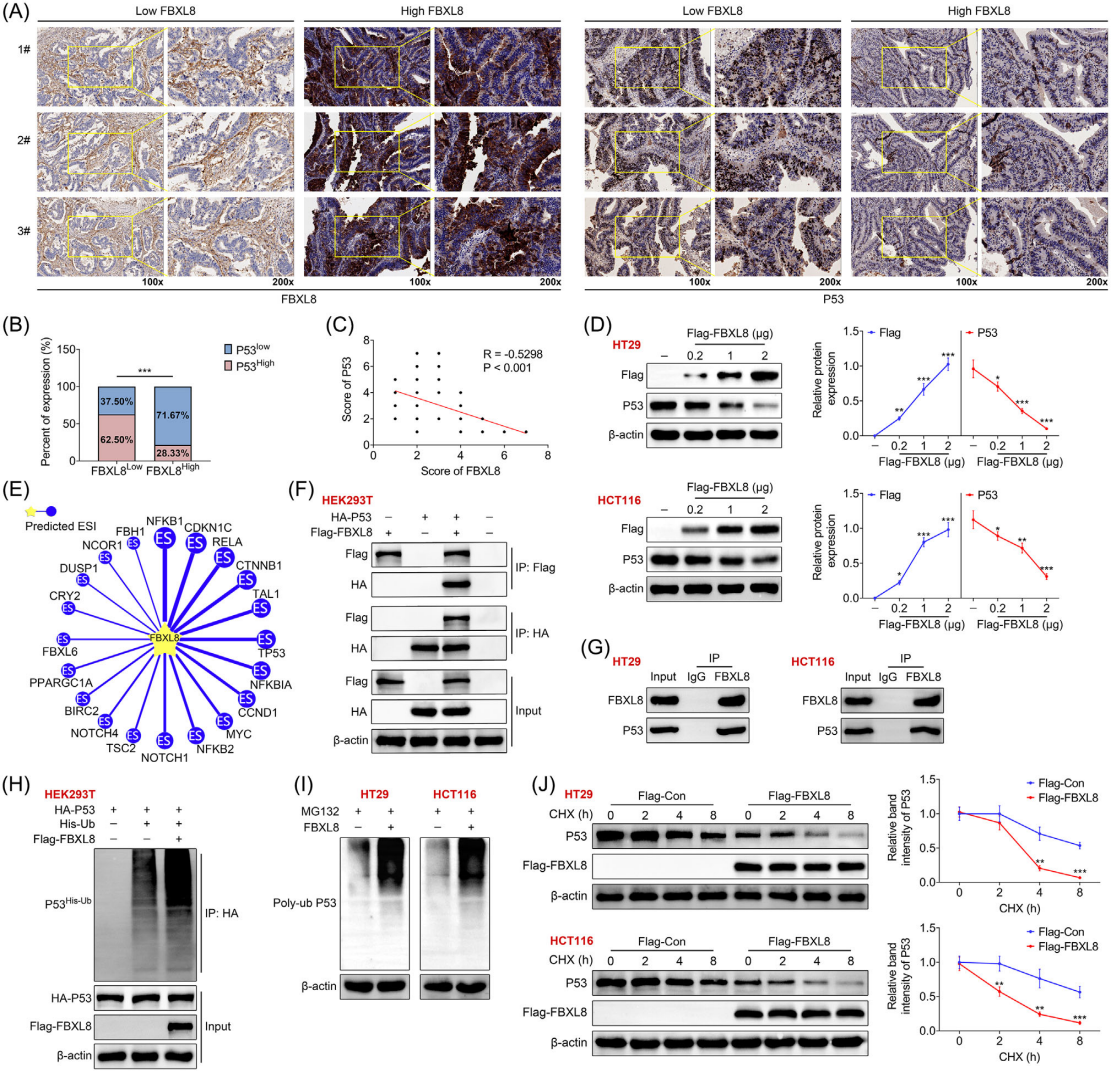
FBXL8 promotes ubiquitination of p53. (A) Representative immunohistochemical images showing FBXL8 and p53 in low FBXL8 and high FBXL8 tissues. (B) The expression percentage of high p53 in low and high FBXL8 tissues. (C) Correlation analysis of FBXL8 and p53 in immunohistochemical score. (D) HT29 and HCT116 cells were treated with flag‐Con or flag‐FBXL8 plasmid (200 ng, 1 μg and 2 μg). Expression levels of FBXL8 and p53 in HT29 and HCT116 cells. (E) Interaction network for proteins identified in association with FBXL8. UbiBrowser database predicts that FBXL8 deubiquitinated p53 protein. (F) HEK293T cells were transfected with Flag‐tagged FBXL8 or HA‐tagged p53, or both for 48 h. Then cells were stained antibodies against Flag‐tag or HA‐tag. The immunoprecipitated proteins were analysed by western blotting. (G) Immunoprecipitation was performed with FBXL8 and IgG antibodies, and western blotting was used to detect the expression levels of FBXL8 and p53. (H) HEK293T cells were transfected with HA‐tagged p53 and/or Flag‐tagged FBXL8 for 48 h. Then cells were stained antibodies against HA tag. The immunoprecipitated proteins were analysed by western blotting. (I) The ubiquitination level of p53 in transfected colorectal cancer (CRC) cells was examined by treating them with 20 μM of MG132 for 3 h. Cells were then mixed with conjugated beads followed by immunoprecipitation with anti‐poly‐ubiquitin antibody. (J) Transfected HCT116 and HT29 cells were treated with 100 μg/mL of CHX. The protein expression of p53 was analysed by western blotting at 0, 2, 4 and 8 h after treatment. The qualification curves of p53 expression in both cells lines at different time points were plotted. *p < .05, **p < .01, ***p < .001 compared with normal group.

We divided CRC tissues into low FBXL8 and high FBXL8 groups and then used immunohistochemistry to analyse the expression levels of FBXL8 and p53 in CRC tissues and scored them (Figure 2A). Overall, 71.67% of tissue samples in high FBXL8 group showed low expression of p53, whereas only 37.50% of samples in low FBXL8 group showed low expression of p53 (Figure 2B). Immunohistochemical score of p53 in CRC tissues was significantly negatively correlated with score of FBXL8 (*R* = −.5298, *p* < .001) (Figure 2C). HT29 and HCT116 cells were transfected with flag‐Con or increasing amount of flag‐FBXL8 plasmid, and it was found that the expression level of FBXL8 protein was significantly enhanced with increasing plasmid concentration, whereas the expression levels of p53 were decreased (Figure 2D). The UbiBrowser database predicted that FBXL8 might degrade P53 protein through deubiquitination (Figure 2E). The results of immunoprecipitation showed that p53 protein could be detected in pull‐down protein by anti‐Flag antibody. Meanwhile, FBXL8 protein was expressed in the samples pulled down by anti‐HA antibody (Figure 2F). HT29 and HCT116 cells were transfected with FBXL8, and the protein was immunoprecipitated with FBXL8 antibody and then incubated with FBXL8 and p53 antibodies. The results showed that p53 was present in the protein immunoprecipitated with FBXL8 antibody (Figure 2G), indicating that FBXL8 binds to p53. Then HEK293T cells transfected with HA‐p53 were simultaneously transfected with His‐Ub or flag, and HA antibody was used for immunoprecipitation. It was found that ubiquitination of p53 was significantly enhanced by overexpression of flag‐FBXL8 (Figure 2H). HT29 and HCT116 cells were transfected with or without FBXL8 plasmid and then treated with MG132, a proteasome inhibitor, and the results showed that p53 ubiquitination levels were increased after overexpression of FBXL8, suggesting that FBXL8 promotes P53 ubiquitination (Figure 2I). In order to clarify the effects of FBXL8 on the stability of p53, CHX chase assay results showed that the protein level of p53 in flag‐FBXL8 group was decreased in time‐dependent manner compared with flag‐Con group (Figure 2J), indicating that FBXL8 accelerated the degradation of p53. In conclusion, FBXL8 can reduce the stability of p53 through ubiquitination.

### 3.3 FBXL8 promoted ubiquitination and degradation of P53 depending on the F‐box and LRR regions

**FIGURE 3:**
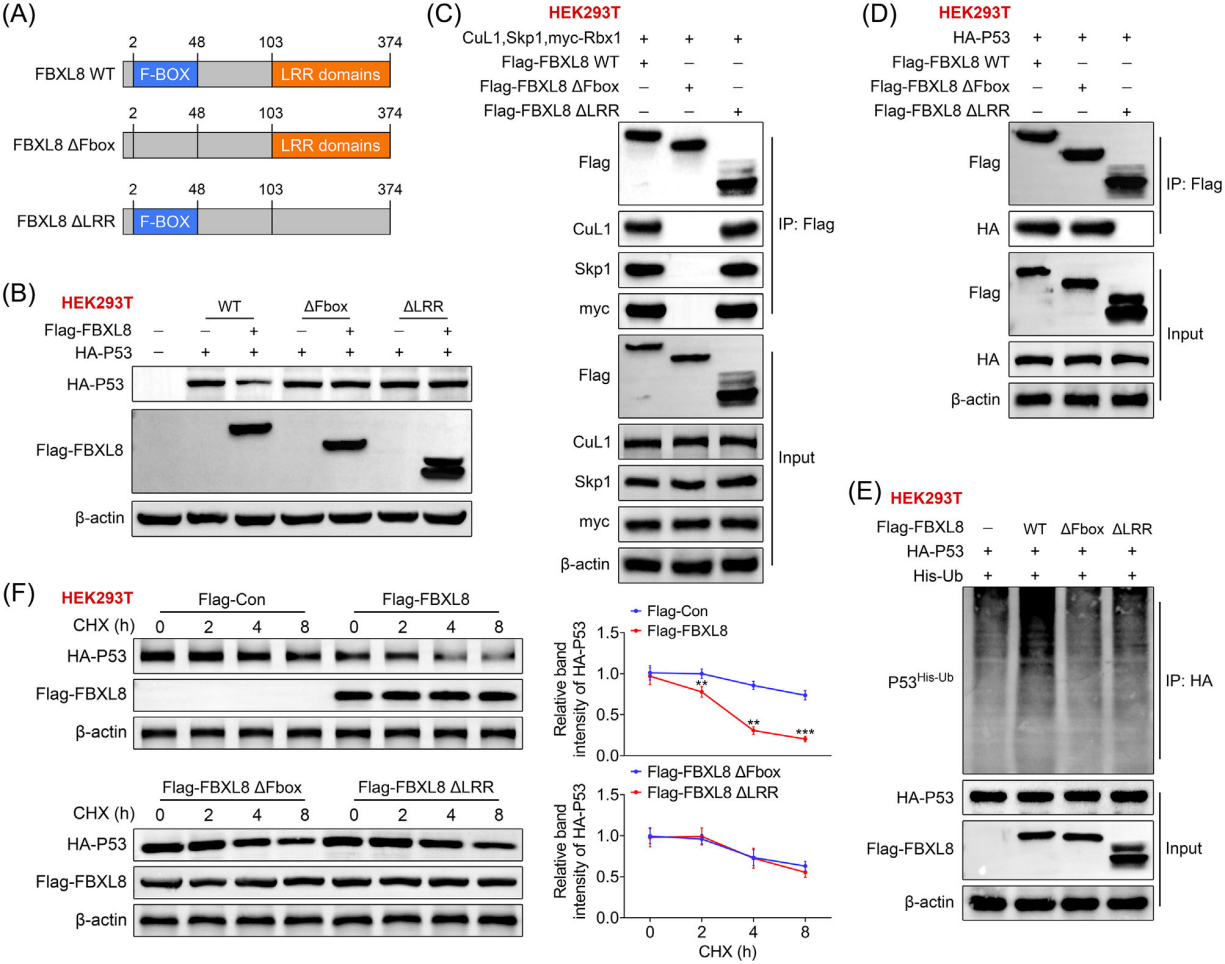
The F‐box and leucine‐rich repeat (LRR) domains of FBXL8 protein play an important role in regulating ubiquitination and degradation of p53. Truncated forms of FBXL8 were constructed based on its functional domains. (A) Construction of full‐length FBXL8 protein, FBXL8 lacking the F‐Box domain (FBXL8 ΔFbox) and FBXL8 lacking the C‐terminal LRR (FBXL8 ΔLRR). (B) HEK293T cells were transfected with Flag‐tagged wild‐type FBXL8, Fbox or LRR mutants together with HA‐p53. Ubiquitylated p53 was detected using an anti‐HA antibody. (C) HEK293T cells expressed SCF complex components (cul1, skp1 and myc‐rbx1) and Flag‐FBXL8 wt, Flag‐FBXL8 ΔFbox or Flag‐FBXL8 ΔLRR plasmids, immunoprecipitation was performed with anti‐Flag beads, and western blotting was performed as shown. (D) HEK293T cells expressed HA‐p53 and Flag‐FBXL8 wt, Flag‐FBXL8 ΔFbox or Flag‐FBXL8 ΔLRR plasmids, and immunoprecipitation was performed with anti‐Flag beads and western blotting as shown. (E) HEK293T cells expressed HA‐p53 and Flag‐FBXL8 wt, Flag‐FBXL8 ΔFbox or Flag‐FBXL8 ΔLRR plasmid, and immunoprecipitation was performed with anti‐HA beads and western blots as shown. (F) HEK293T cells were treated with HA‐p53 and Flag‐FBXL8 wt, Flag‐FBXL8 ΔFbox or Flag‐FBXL8 ΔLRR plasmids, CHX (100 μg/mL) for different times, and the corresponding protein levels were measured by western blotting. **p < .01, ***p < .001 compared with flag‐Con group.

To determine which domain of FBXL8 mediated the degradation of p53, we constructed FBXL8 ΔFbox and FBXL8 ΔLRR mutants (Figure 3A). We observed that FBXL8 truncations abolished the interaction with p53 (Figure 3B), suggesting that only full‐length FBXL8 has an effect on ubiquitination of p53. Anti‐Flag antibody was used to pull‐down protein, and immunoprecipitation assay showed that clu1, skp1, myc‐rbx1 (SCF component) bound to FBXL8 WT and FBXL8 ΔLRR mutants. However, there was no binding between SCF components and FBXL8 ΔFbox (Figure 3C), indicating that Fbox region is important for the binding between FBXL8 and SCF. In addition, FBXL8 ΔLRR mutants abolished p53 ubiquitination, indicating that LRR is essential for ubiquitination of p53 (Figure 3D). Immunoprecipitation assay showed that only full‐length FBXL8 could promote ubiquitination of p53, but not FBXL8 ΔFbox and FBXL8 ΔLRR (Figure 3E). To clarify the effects of FBXL8 wt, FBXL8 ΔFbox or FBXL8 ΔLRR on p53 stability, CHX chase assay was conducted and the results showed that flag‐FBXL8 promoted p53 degradation. However, FBXL8 ΔFbox and FBXL8 ΔLRR have no significant influence on the stability of p53 (Figure 3F).

### 3.4 FBXL8 promoted CRC cell proliferation, migration and stem‐cell‐like properties

**FIGURE 4:**
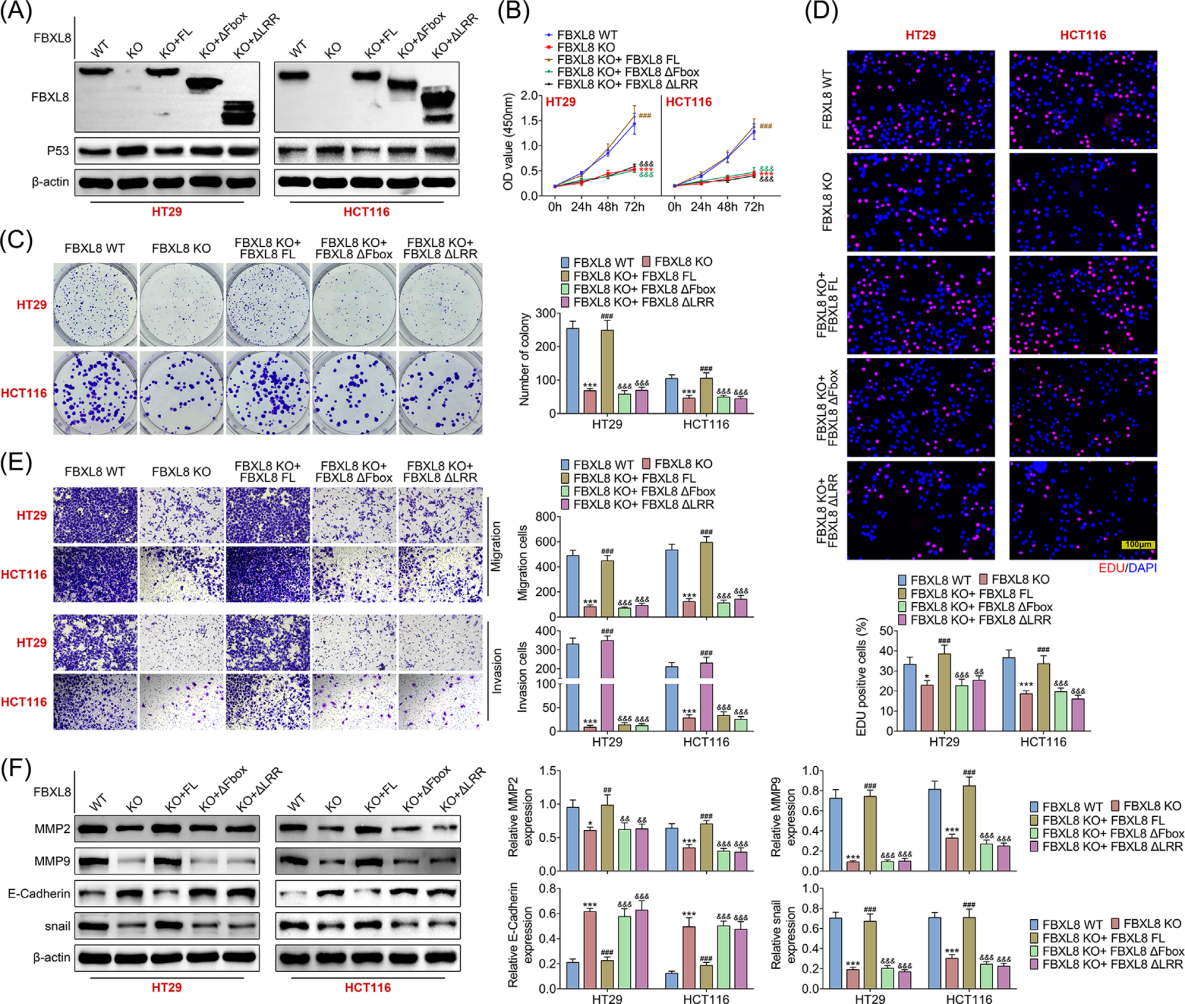
FBXL8 promotes colorectal cancer (CRC) cell proliferation and migration. After FBXL8 KO or FBXL8 ΔFbox, FBXL8 ΔLRR were constructed in CRC cells, and the protein expression levels of FBXL8 and p53 were detected by western blotting (A); cell viability was measured by CCK8 (B); cloning by crystal violet staining (C); cell proliferation was measured by EDU (D); transwell measured migration and invasion ability of cells (E), and western blotting detected migration related indicators (MMP2, MMP9) and expression of *E*‐cadherin, snail (F). *p < .05, ***p < .001 compared with FBXL8 WT group; ^###^ *p* < .001 compared with FBXL8 KO group; ^&&&^ *p* < .001 compared with FBXL8 KO + FBXL8 FL group.

FBXL8 plays an important role in the pathological mechanism of a few tumours.^21^, ^22^ We explored the effect of FBXL8 on the pathological process of CRC. FBXL8 KO or FBXL8 ΔFbox, FBXL8 ΔLRR plasmids were transfected into HT29 and HCT116 cells, and the level of p53 was detected by western blotting. It was found that the expression of p53 in FBXL8 KO group was significantly higher than that in FXBL8 WT group. Compared with the FBXL8 KO group, only the FBXL8 KO + FL group had a lower expression level of p53, but not the FBXL8 KO + ΔFbox or FBXL8 ΔLRR group (Figure 4A). Cell viability was detected by CCK8, and the viability of cells was lower in FBXL8 KO group showed compared with FBXL8 WT group (Figure 4B). In addition, compared with FBXL8 KO group, only FBXL8 KO + FL group, but not FBXL8 KO + ΔFbox or FBXL8 ΔLRR group, showed significantly enhanced cell proliferation ability (Figure 4B). In‐line with cell activity, FBXL8 KO group showed fewer colonies than FBXL8 WT group (Figure 4C). Compared with FBXL8 KO group, only FBXL8 KO + FL group, but not FBXL8 KO + ΔFbox or FBXL8 ΔLRR group, showed significantly increased number of cell clonogenesis (Figure 4C). The EDU experiment further confirmed that after FBXL8 was knocked out, HT29 and HCT116 cells showed significantly reduced proliferation ability, and only full‐length FBXL8 knockout affected cell proliferation ability (Figure 4D). Transwell assay was used to detect the migration and invasion abilities of cells, and it was found that there were fewer migratory cells in the FBXL KO group than in the FBXL8 WT group (Figure 4E). The number of migrated cell and invaded cells in the FBXL8 KO + FBXL8 FL group was significantly increased compared with the FBXL8 KO group (Figure 4E). There was no statistical difference between the FBXL8 KO + ΔFbox or FBXL8 ΔLRR group and the FBXL8 KO group (Figure 4E). In addition, the expression levels of migration‐related markers metalloproteinases (MMP2 and MMP9), epithelial mesenchymal transformation related indicator (*E*‐cadherin) and snail were detected. Compared with KBXL8 WT group, KBXL8 KO group showed down‐regulated protein levels of MMP2, MMP9 and snail, and the up‐regulated protein level of *E*‐cadherin (Figure 4F). Compared with KBXL8 KO group, KBXL8 KO + FL group showed up‐regulated protein levels of MMP2, MMP9 and snail, and down‐regulated protein levels of *E*‐cadherin. However, there was no statistical difference in these indicators between the FBXL8 KO + ΔFbox group and the FBXL8 ΔLRR group (Figure 4F). These data suggest that only full‐length FBXL8 knockout can inhibit the proliferation, invasion and migration of HT29 and HCT116 cells.

**FIGURE 5:**
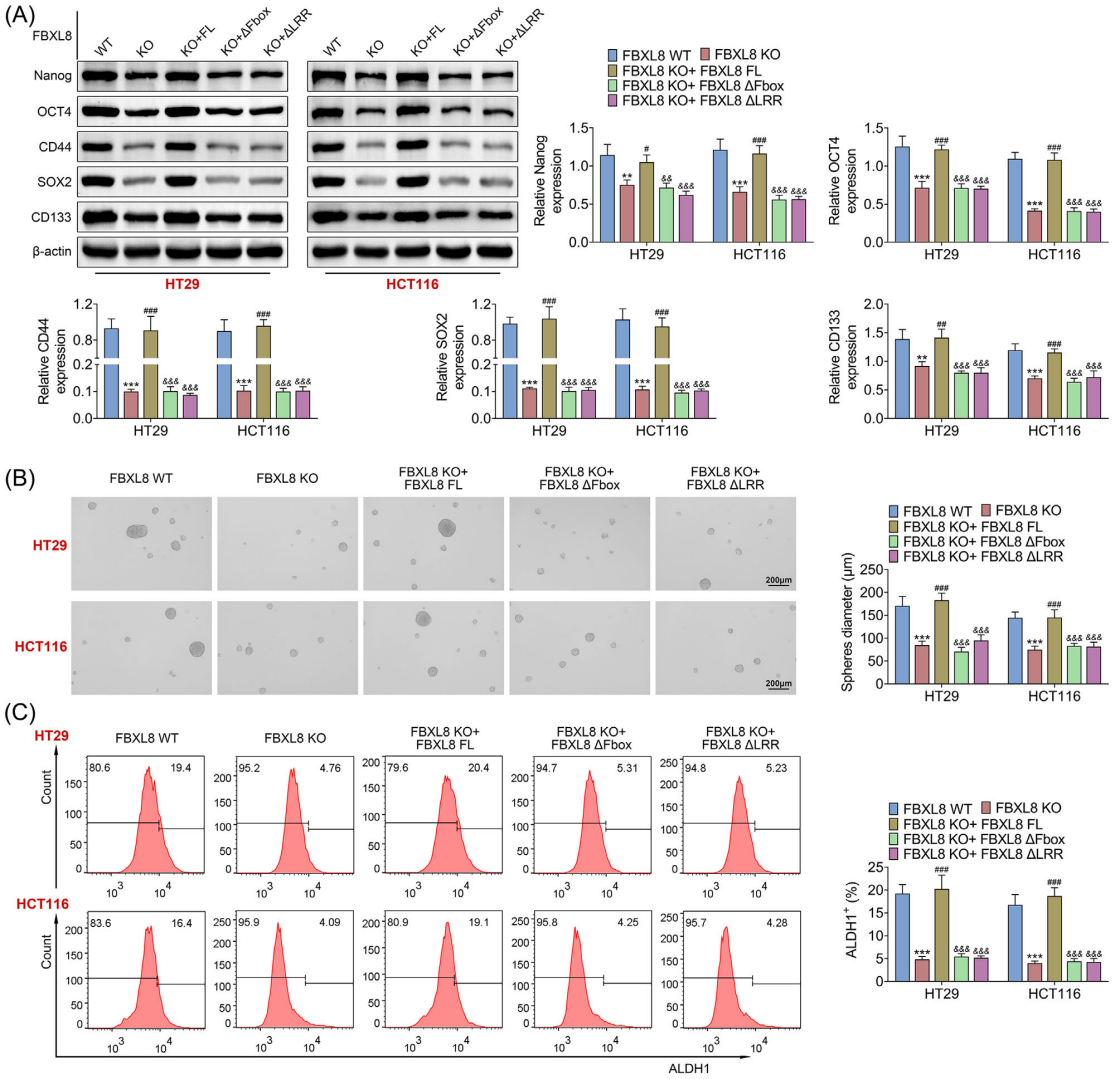
FBXL8 promotes stem‐cell‐like properties of colorectal cancer (CRC) cells. After FBXL8 KO or FBXL8 ΔFbox, FBXL8 ΔLRR were constructed in CRC cells, and the protein expression levels of Nanog, OCT4, CD44, SOX2 and CD133 related to stem‐cell characteristics were detected by western blotting (A); sphere cell formation was performed (B). The expression levels of ALDH1 were detected by flow cytometry (C). **p < .01, ***p < .001 compared with FBXL8 WT group;^#^ *p* < .05, ^##^ *p* < .01, ^###^ *p* < .001 compared with FBXL8 KO group;^&&^ *p* < .01, ^&&&^ *p* < .001 compared with FBXL8 KO + FBXL8 FL group.

Stem‐cell‐like properties play an important role in tumour progression.^31^, ^32^, ^33^ Western blotting showed that compared with the FBXL8 WT group, protein expression levels of stem‐cell characteristics related indicators (Nanog, OCT4, CD44, SOX2 and CD133) were significantly reduced in the FBXL8 KO group (Figure 5A). Compared with FBXL8 KO group, Nanog, OCT4, CD44, SOX2 and CD133 were up‐regulated in KBXL8 KO + FL group (Figure 5A). Sphere‐forming experiments showed that compared with the FBXL8 WT group, the diameter of spherical cells was significantly reduced in the FBXL8 KO group (Figure 5B). Compared with the FBXL8 KO group, the diameter of spherical cells was up‐regulated in the KBXL8 KO + FL group (Figure 5B). Flow cytometry results showed that ALDH1^+^ cells were significantly reduced in the FBXL8 KO group compared to the FBXL8 WT group (Figure 5C). Compared with FBXL8 KO group, KBXL8 KO + FL group showed a significant increase in ALDH1^+^ cells (Figure 5C). In addition, there were no statistically significant differences in stem‐cell characteristics, spherical cell diameter and ALDH1^+^ cells in FBXL8 KO + ΔFbox or FBXL8 ΔLRR group compared with FBXL8 KO group. These results suggest that full‐length FBXL8 promotes stem‐like properties of CRC cells, but not FBXL8 KO + ΔFbox or FBXL8 ΔLRR.

### 3.5 FBXL8 knockout inhibited CRC growth and liver metastasis in vivo

**FIGURE 6:**
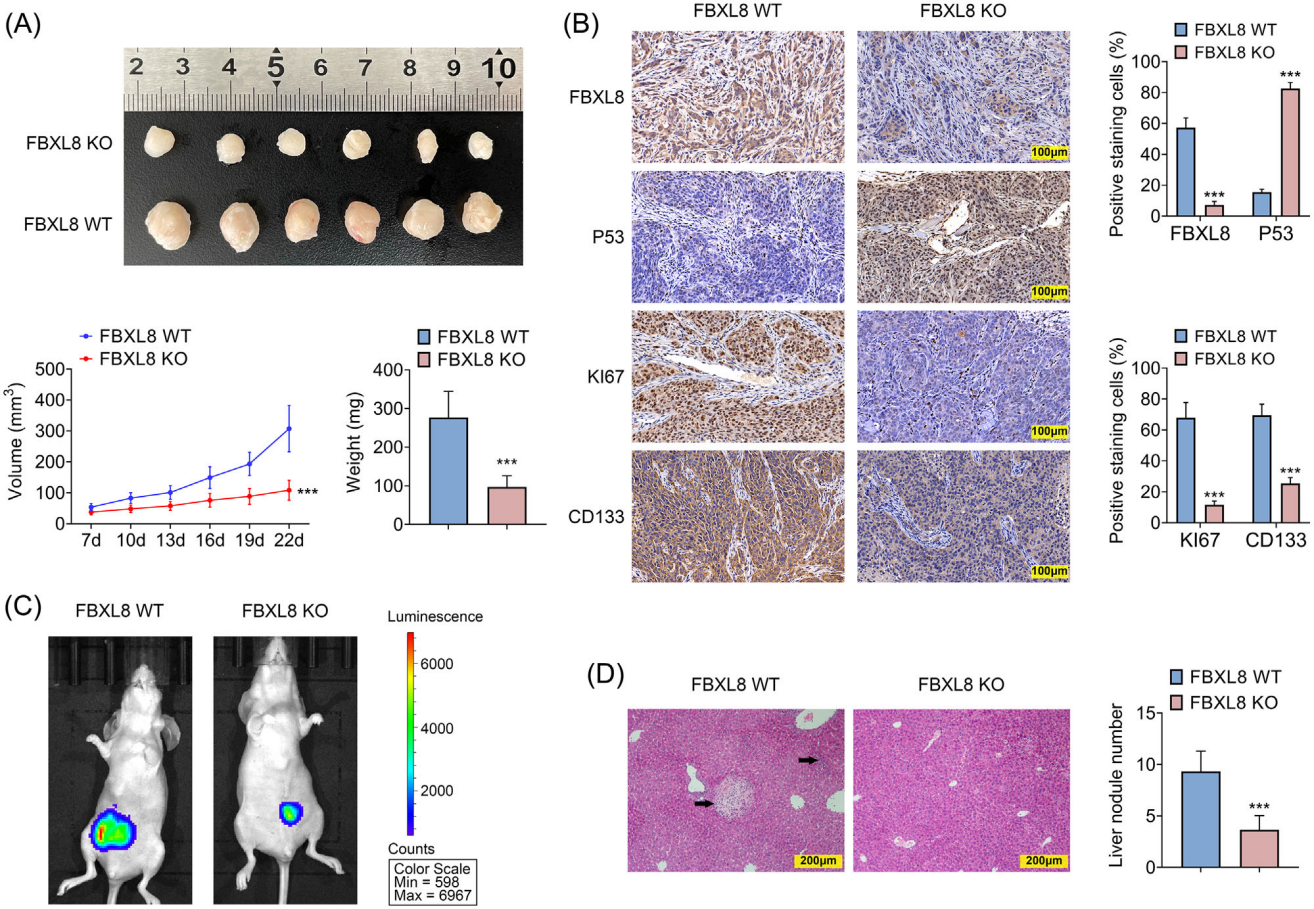
FBXL8 knockout inhibits colorectal cancer (CRC) growth and liver metastasis in vivo. Twelve BALB/C nude mice were divided into two groups with six mice in each group. The xenograft tumour model was established by subcutaneous injection of HT29 (5 × 10^5^ cells/mouse). (A) Tumour volume and tumour level in the FBXL8 WT and FBXL8 KO groups. (B) Immunohistochemical representative images showed the expressions of FBXL8, p53, Ki67 and CD133 in FBXL8 WT and FBXL8 KO groups. (C and D) Twelve BALB/C nude mice were divided into two groups with six mice in each group. HT29 cells (5 × 10^5^ cells/mouse) were injected into the spleen of mice to establish tumour metastasis model. Hepatic metastasis was measured by IVIS Lumina imaging system (C). (D) Liver tissue of mice was stained with haematoxylin–eosin (H&E) to determine the status of lymph nodes with hepatic metastasis. ***p < .001 compared with FBXL8 WT group.

To explore the effect of FBXL8 on pathological progression of CRC, in vivo assay was performed. The tumour volume and tumour weight were reduced in the FBXL8 KO group compared to the FBXL8 WT group (Figure 6A). The expression levels of Ki67 and CD133 were decreased, and p53 expression was up‐regulated in FBXL8 KO group compared with FBXL WT group (Figure 6B). FBXL8 knockout HT29 cells were injected into the spleen of mice to establish tumour metastasis model. IVIS Lumina imaging System detected reduced liver metastasis of CRC in the FBXL8 KO group (Figure 6C). Haematoxylin–eosin staining showed that the number of liver metastatic lymph nodes in FBXL8 KO group was significantly reduced (Figure 6D). These results suggested that FBXL8 is involved in the pathological mechanism of CRC and the process of liver metastasis.

### 3.6 Down‐regulation of p53 expression can inhibit the anticancer effect of FBXL8 knockout

**FIGURE 7:**
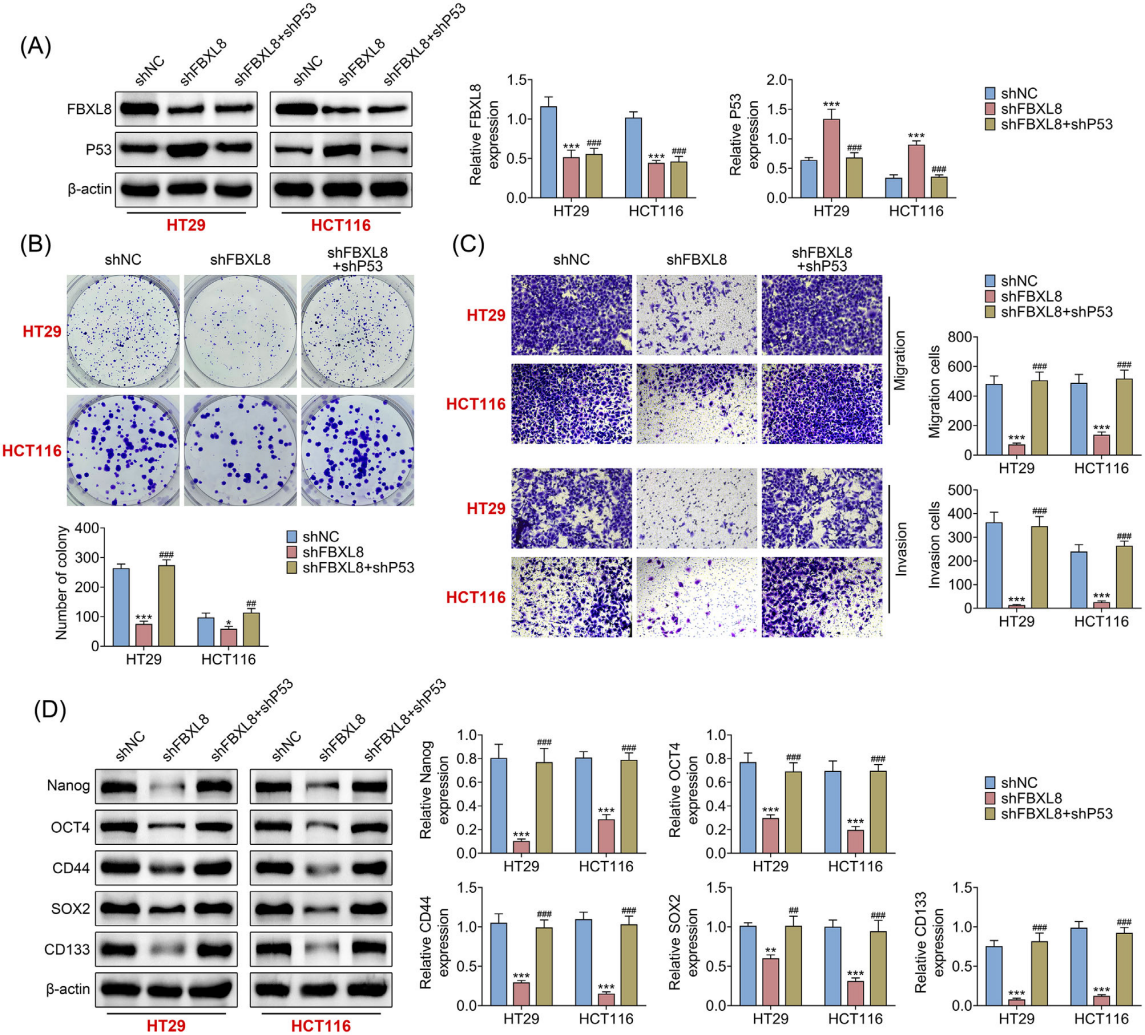
Down‐regulation of P53 expression can inhibit the anticancer effect of FBXL8 knockout. FBXL8 and/or P53 were knocked out in HT29 and HCT116 cells, and the protein expression levels of FBXL8 and P53 were detected by western blotting (A), cloning by crystal violet staining (B), transwell measured the migration and invasion ability of cells (C) and the protein expression of Nanog, OCT4, CD44, SOX2 and CD133 was detected by western blotting (D). **p < .01, ***p < .001 compared with shNC group; ^##^ *p* < .01, ^###^ *p* < .001 compared with shFBXL8 group.

P53 is a known tumour suppressor that plays an important role in tumour formation.^34^, ^35^, ^36^ FBXL8 and/or p53 were knocked out in HT29 and HCT116 cells to explore the effect of FBXL8 on p53. The knockout efficiency of FBXL8 and p53 was verified by western blotting (Figure 7A). Compared with the shNC group, cell colony formation was lower in the shFBXL8 group, which could be reversed after p53 downregulation (Figure 7B). Transwell experiments showed that compared with the shNC group, shFBXL8 group showed fewer migration and invasion cells, and this effect was rescued after p53 knockdown (Figure 7C). In addition, western blotting showed that the expressions of stem‐cell characteristics related indicators (Nanog, OCT4, CD44, SOX2 and CD133) in the shFBXL8 group were significantly reduced compared with the shNC group, and when p53 was down‐regulated, the expression levels of these indicators were recovered (Figure 7D).

## 4 DISCUSSION

Our study found that FBXL8 is highly expressed in CRC. Mechanism studies have shown that FBXL8 can degrade tumour suppressor gene p53 through ubiquitination, which plays an important role by binding F‐box region to SCF and LRR region to target protein p53. In addition, FBXL8 knockout inhibited the proliferation and stem‐cell‐like characteristics of CRC cells.

First, the up‐regulated expression of FBXL8 was found in CRC through database analysis, and the high expression of FBXL8 in CRC was further verified by our clinical samples. CRC was further divided into nmCRC and mCRC according to the presence or absence of liver metastasis, and FBXL8 was found to be highly expressed in mCRC, suggesting that FBXL8 may be associated with poor prognosis in CRC patients. Then, we divided CRC patients into high FBXL8 and low FBXL8 groups and found that CRC patients with high FBXL8 had lower overall survival rate. These data suggested that FBXL8 may be involved in the pathogenesis of CRC.

As a well‐known tumour suppressor, p53 plays a particularly important role in tumour formation.^36^, ^37^, ^38^ By dividing CRC tissue into high and low FBXL8 groups, our results showed that p53 expression was significantly lower in the high FBXL8 group. There was a significant negative correlation between p53 and FBXL8 expression level, suggesting that FBXL8 may participate in CRC process by regulating p53. To test this hypothesis, we first transfected FBXL8 plasmid into HT29 and HCT116 cells and found that the expression of p53 was decreased with increase of FBXL8 plasmid concentration. We further found that FBXL8 can bind to p53 and degrade p53 through ubiquitination, which was verified by co‐immunoprecipitation and ubiquitination detection.

The F‐box component of E3 ligase is involved in tumour progression.^11^, ^39^, ^40^, ^41^ To clarify the region where FBXL8 acts on p53, we constructed plasmids with truncation of the Fbox and LRR regions. Immunoprecipitation showed that only FBXL8 WT ubiquitinated p53. These results suggest that both Fbox and LRR were required for the ubiquitination of p53. As a member of Fbox E3 ligase, FBXL8 binds to other components of E3 ligase, that is it specifically binds to substrates to induce ubiquitination. We found that SCF components could not be detected in the anti‐Flag protein pulled down after truncation of Fbox region of FBXL8, suggesting that the Fbox region of FBXL8 is the binding site for binding to other E3 ligase. To clarify which region of FBXL8 is the binding region of the substrate, our study found that when HEK293T was transfected with HA‐p53 and FBXL8 WT, FBXL8 Fbox or FBXL8 LRR truncations, only FBXL8 LRR truncation blocked the ubiquitination of p53, suggesting that the LRR region of FBXL8 binds to p53. Further, overexpression of FBXL8 can promote ubiquitination of p53 and reduce the stability of p53, which is consistent with previous reports that Fbox members of the E3 ligase family reduce stability of tumour suppressor through ubiquitination.^11^

Members of the E3 ligase Fbox have been reported to promote the development and progression of a variety of tumours.^11^, ^42^, ^43^, ^44^ To elucidate the effect of FBXL8 on the pathogenesis of CRC, FBXL8 or Fbox or LRR regions of FBXL8 were knocked out in HT29 and HCT116 cells. Knockout of FBXL8 decreased the cell viability, colony formation, proliferation, migration and expression of metalloproteinase and snail compared with FBXL8 WT group. Compared with FBXL8 KO + FBXL8 FL group, Fbox and LRR region knockout made the results consistent with the above trend. Compared with FBXL8 KO group, Fbox and LRR knockout did not affect cell viability, proliferation and migration. These data suggest that only FBXL8 FL affects pathological processes of CRC, and that Fbox and LRR regions are essential for the FBXL8 function in CRC. The role of stem‐cell‐like properties in tumour formation is receiving increasing attention. After FBXL8 was knocked down, western blotting showed that the indicators related to stem‐cell‐like characteristics were down‐regulated, the cell sphere diameter was shortened, and the indexes related to stem‐cell‐like characteristics ALDH1 were down‐regulated. Similarly, knockout in the Fbox and LRR regions did not affect stem‐cell‐like specificity compared with FBXL8 KO group. These results demonstrate that high expression of FBXL8 promoted CRC cell proliferation, migration and stem‐cell‐like properties, dependent on its Fbox and LRR regions. In vivo study showed that FBXL8 knockout inhibited the CRC tumour growth in mice. In addition, by establishing a mouse model of liver metastasis, the results indicated that there were fewer liver metastasis and lymph nodes metastasis in the FBXL8 KO group. These results suggest that FBXL8 knockout can inhibit the proliferation and liver metastasis of CRC tumours. In order to explore the effect of FBXL8 on p53, FBXL8 and/or p53 were knocked out in HT29 and HCT116 cells. After FBXL8 was knocked out, the expression of p53 was increased. In addition, FBXL8 knockout also decreased the tumour cell colony formation and inhibited the cell migration and invasion, as well as down‐regulated the expression of stem‐cell‐related indicators, whereas 53 knockout had the opposite effects. These results suggest that down‐regulation of p53 expression can inhibit FBXL8 inhibition.

There are still some limitations in our study. First, there are two bands in the blotting of FBXL8 ΔLRR in Figure 3. We analysed that the possible reason is that the existing technology is not enough to completely knockout FBXL8 ΔLRR. Second, we did not pay attention to the expression of FBXL8 on HT29 and HCT116 in KRAS mutation state, which will be explored in the future studies (Figure S1, dummy citation).

Our study suggests that FBXL8 is up‐regulated in CRC and reduces the stability of p53 by binding to SCF components through its Fbox region and by binding to p53 through its LRR region to promote the ubiquitination of p53, thereby contributing to the pathological mechanism of CRC by promoting cell proliferation, migration and stem‐cell‐like properties.

## CONFLICT OF INTEREST STATEMENT

The authors declare no conflicts of interest.

## STATEMENT OF INFORMED CONSENT

Written informed consent was obtained from a legally authorized representative(s) for anonymized patient information to be published in this article.

## Supporting information

Supporting Information
