# Supplementary figures and images for: SCF‐FBXL8 contributes to liver metastasis and stem‐cell‐like features in colorectal cancer cells by mediating ubiquitination and degradation of TP53
Source: Clin Transl Med. 2023 Feb 28;13(3):e1208. doi: 10.1002/ctm2.1208 (PMC9975457; doi:10.1002/ctm2.1208)

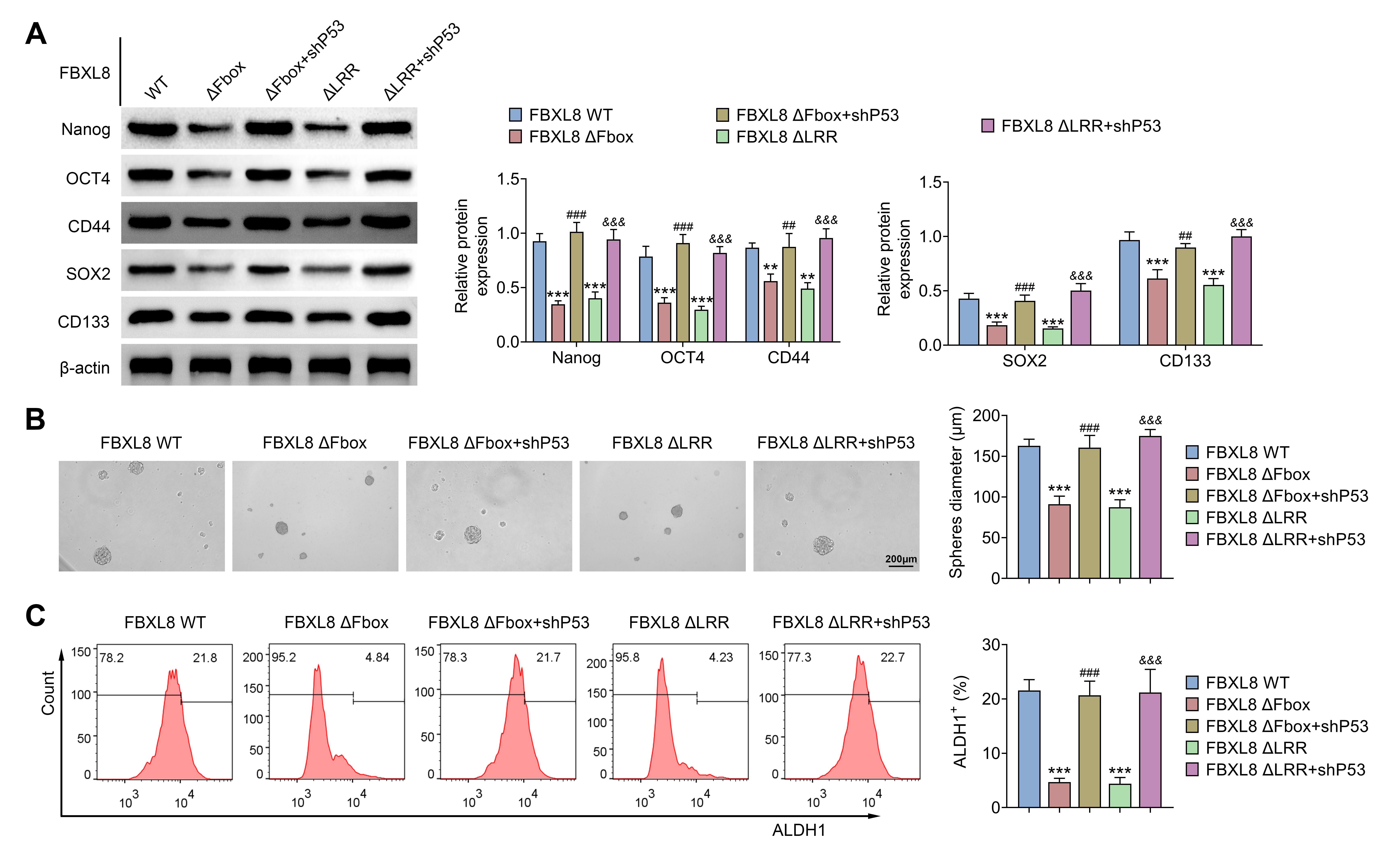

Supplement: Supplementary file 1 — Supporting Information [file CTM2-13-e1208-s001.jpg]
